# Bamboo polyphenols protects against *Escherichia coli*-induced liver injury in broilers by attenuating inflammatory responses

**DOI:** 10.1016/j.psj.2026.107402

**Published:** 2026-07-05

**Authors:** Yuzhang Chen, Jie Zeng, Lu Liu, Ning Li, Mengyao Liu, Kaicheng Zhang, Zexi Zhang, Bingjie Zou, Xiaojuan Chi, Song Wang

**Affiliations:** a Key Laboratory of Animal Pathogen Infection and Immunology of Fujian Province, College of Animal Sciences, Fujian Agriculture and Forestry University, Fuzhou, 350002, China; b Key Laboratory of Fujian-Taiwan Animal Pathogen Biology, College of Animal Sciences, Fujian Agriculture and Forestry University, Fuzhou, 350002, China; c Joint Laboratory of Animal Pathogen Prevention and Control of Fujian-Nepal, College of Animal Sciences, Fujian Agriculture and Forestry University, Fuzhou, 350002, China; d Fujian Sunvet Biotechnology Co., Ltd, Nanping, 354100, China

**Keywords:** Bamboo polyphenols, Antibacterial activity, Anti-inflammatory activity, NF-κB signaling pathway, MAPK signaling pathway

## Abstract

Avian colibacillosis, caused by avian pathogenic *Escherichia coli* (APEC), is a highly prevalent disease worldwide and leads to substantial economic losses in the poultry industry annually. Despite the well-documented bioactivities of various plant polyphenols, the therapeutic potential of bamboo polyphenols (BP) against APEC infection remains largely unexplored. In this study, we investigated the antibacterial and anti-inflammatory effects of BP both *in vitro* and *in vivo*. The results demonstrated that BP inhibited the growth and killed APEC at relatively low concentrations in bacteriostatic and bactericidal assays. Liver histopathological examination and bacterial load determination revealed that treatment with BP reduced inflammatory cell infiltration and significantly decreased bacterial burden in liver tissue. Moreover, BP markedly downregulated the mRNA expression levels of inflammatory cytokines, chemokines, and pro-inflammatory markers, and suppressed the phosphorylation of p65, p38, and ERK1/2 both *in vitro* and *in vivo*. Additionally, BP inhibited LPS-induced ROS expression *in vitro*. These findings show that BP exerts antibacterial and anti-inflammatory effects by inhibiting bacterial growth, suppressing the NF-κB and MAPK pathways, and reducing ROS levels. This offers a new theoretical basis for controlling bacterial diseases in livestock and poultry, as well as a novel strategy against bacterial resistance.

## Introduction

Avian colibacillosis is an infectious disease caused by avian pathogenic *Escherichia coli* (APEC) (Kathayat et al., 2021), which manifests as both localized and systemic infections in poultry. Localized forms include peritonitis, salpingitis, omphalitis, orchitis, osteomyelitis/synovitis, panophthalmitis, and coliform cellulitis. In contrast, systemic infections—such as colisepticaemia and coligranulomatosis—are associated with high morbidity and mortality (Dziva et al., 2008).

APEC primarily infects poultry through the respiratory tract, although alternative routes of entry, including the oral, nasal, and cloacal pathways, have also been documented. Under normal conditions, APEC colonizes the mucosal surfaces of the gastrointestinal, respiratory, and reproductive tracts without inducing clinical disease. However, the bacterium can act as a primary pathogen or induce systemic infections, particularly when birds are co-infected with other pathogens or exposed to environmental stressors (Clermont et al., 2019; Denamur et al., 2021). Transmission occurs via the fecal-oral route or through aerosolized particles, with contaminated feed and water serving as key vehicles for bacterial dissemination. Vertical transmission through infected eggs has also been reported (Sarowska et al., 2019; Kot et al., 2019; Azam et al., 2020).

Colonization of the respiratory tract is a critical step in APEC pathogenesis and is mediated by an array of virulence factors, including adhesins, serum resistance genes, iron acquisition systems, hemolysins, toxins, invasion-associated proteins, and the CS_2_ subsystem (Alber et al., 2021; Denamur et al., 2021). Pathogenic *Escherichia coli* (*E. coli*) are broadly classified into two major groups: intestinal pathogenic *E. coli* (IPEC) and extraintestinal pathogenic *E. coli* (ExPEC), with APEC belonging to the latter (Kabiswa et al., 2018; Kurnia et al., 2018; Kimura et al., 2021). Within the ExPEC group, APEC isolates frequently express serotypes O1, O2, O18, or O78 (Silveira et al., 2016). Notably, more than 80% of APEC infections are attributed to serotypes O1, O2, and O78, among which O78 is particularly predominant worldwide (Jamali et al., 2024).

Poultry meat represents a major source of animal protein essential for meeting global nutritional demands. The widespread transmission of APEC has led to substantial economic losses worldwide, primarily resulting from reduced egg production and increased mortality rates. Current control strategies against APEC predominantly rely on the use of various antibiotics (Barbieri et al., 2017). However, the emergence of antibiotic-resistant APEC strains has become a significant global health concern, adversely affecting both animal health and food safety (Guerra et al., 2018; Xu et al., 2019; Thomrongsuwannakij et al., 2020). APEC has demonstrated resistance to nearly all classes of antibiotics, with susceptibility largely retained only to carbapenems, imipenem, and streptomycin. These findings underscore the urgent need for comprehensive strategies to combat antibiotic resistance in APEC and safeguard public health.

Polyphenols are well-recognized natural antioxidants and secondary metabolites produced by plants (Manach et al., 2004). Based on their chemical structures, polyphenols can be classified into two major categories: flavonoids and non-flavonoids (Xiao et al., 2013). Flavonoids are further subdivided into flavones, flavonols, flavanones, and isoflavones (Xiao et al., 2012), whereas non-flavonoids mainly include stilbenes, chalcones, anthraquinones, ellagitannins, ellagic acid, and phenolic acids. These compounds are abundant in various plant-based foods, such as fruits, vegetables, cereals, olives, cocoa, tea, coffee, and wine (Cardona et al., 2013). Representative examples include anthocyanins in brightly colored fruits and vegetables, isoflavones in soybeans, catechins in grapes and wine, proanthocyanidins in cocoa, resveratrol in wine, and naringenin and hesperidin in citrus fruits.

Polyphenols have been demonstrated to exhibit a wide range of bioactivities, including anti-inflammatory, antibacterial, antioxidant, anticancer, anti-adipogenic, antidiabetic, and neuroprotective effects (Cassidy et al., 2011; Chiva-Blanch et al., 2013; Ahtesh et al., 2020). Among these, the antibacterial activity has attracted considerable interest due to its potential to combat drug-resistant bacteria that are insensitive to conventional antibiotics. Specifically, flavonoids exert antibacterial effects through three primary mechanisms: direct bactericidal action, synergistic enhancement of antibiotic activity, and attenuation of bacterial pathogenicity (Cushnie et al., 2011). A study investigated the protective effect of red wine polyphenol extract against *E. coli*-induced enterocyte cytotoxicity. The extract directly interacts with bacterial exotoxins and epithelial cells in a concentration-dependent manner, thereby blocking toxic effects and significantly reducing cell death (Nunes et al., 2019). Another study demonstrated that Portulaca oleracea, which is rich in phenolic acids, effectively ameliorates intestinal barrier disruption and inflammation, modulates the gut microbiota, and exerts therapeutic effects against ulcerative colitis by inhibiting the nuclear factor kappa-light-chain-enhancer of activated B cells (NF-κB) signaling pathway (Li et al., 2025). In a lipopolysaccharide (LPS)-induced inflammatory model using RAW264.7 macrophages, Cornus officinalis polyphenol extract was shown to reduce pro-inflammatory markers by suppressing v-akt murine thymoma viral oncogene homolog (AKT) phosphorylation (Najjar et al., 2021). Collectively, these findings indicate that polyphenolic compounds possess significant antibacterial and anti-inflammatory properties.

Despite this growing body of evidence, no studies to date have investigated the effects of bamboo polyphenols—polyphenolic compounds extracted from processed bamboo materials—on systemic diseases caused by APEC infection. Therefore, the present study aims to establish an inflammatory model by infecting white-feathered broilers with APEC, evaluate the antibacterial and anti-inflammatory efficacy of bamboo polyphenols, and explore their potential as a novel antibiotic alternative to address the growing challenge of antibiotic resistance.

## Materials and methods

### Bacterial strains, cell lines and reagents

*E. coli* O1 (CVCC1567) and *Staphylococcus aureus* (CVCC2262) were sourced from National Center for Veterinary Culture Collection (CVCC, Beijing, China). *Salmonella Enteritidis* (CMCC(B) 50335) was obtained from National Center for Medical Culture Collection (CMCC, Beijing, China). *Salmonella Typhimurium* (ATCC14028) was obtained from American Type Culture Collection (ATCC, Manassas, VA, USA). *E. coli* O78 was obtained as previously described (Wang et al., 2021). HD11 cells were obtained from ATCC (Manassas, VA, USA). The cells were cultured in Dulbecco’s Modified Eagle’s Medium (Procell System, Wuhan, China) supplemented with 10% fetal bovine serum (Vazyme Biotech, Nanjing, China) and 100 U/mL penicillin-streptomycin (Beyotime, Shanghai, China), and maintained at 37°C in a humidified atmosphere containing 5% CO_2_.

The following reagents were used in this study: LPS (Sigma, MO, USA); Cell Counting Kit-8 (CCK-8), and Reactive Oxygen Species (ROS) Assay Kit (Beyotime, Shanghai, China); anti-p38, anti-phospho-p38, anti-ERK1/2, anti-phospho-ERK1/2, and β-actin (CST, Massachusetts, USA); anti-p65 (ABclonal, Wuhan, China); anti-phospho-p65 (HUABIO, Hangzhou, China); Bamboo polyphenols (BP) were kindly provided by Fujian Sunvet Biotechnology Co., Ltd (Nanping, China).

### Animal experiment

Twenty-day-old male white-feathered broilers were randomly assigned to four groups (n = 10 per group): the blank group (Mock), the BP-treated group (BP), the *E. coli*-infected group (*E. coli*), and the *E. coli* and BP co-treated group (*E. coli* + BP). Broilers in the BP and *E. coli* + BP groups received 0.4% BP in their drinking water for three consecutive days prior to infection, whereas those in the Mock and *E. coli* groups were given untreated drinking water. This concentration was selected based on the commercially recommended dosage for broiler production, which has been validated through practical application in poultry farms. The estimated daily BP intake was 400 mg/kg body weight. Following the pretreatment period, an inflammatory model was established by intraperitoneal injection of 3.0 × 10^9^ CFU of *E. coli* per bird into broilers in the *E. coli* and *E. coli* + BP groups. Broilers in the Mock and BP groups received an equivalent volume of sterile saline.

### Determination of the MIC and MBC of BP

The minimum inhibitory concentration (MIC) and minimum bactericidal concentration (MBC) of BP were determined using the broth microdilution method in 96-well plates as previously described (Azimzadeh et al., 2025). Briefly, BP was serially two-fold diluted to generate a series of concentration gradients. Then, 100 μL of bacterial suspension was inoculated into 100 μL of LB broth containing varying concentrations of BP, yielding a final bacterial concentration of 5.0 × 10^5^ CFU/mL, and the mixture was incubated statically at 37°C for 24 h. The MIC was defined as the lowest concentration of BP at which the medium remained clear with no visible bacterial growth. For the MBC, 100 μL of LB broth containing BP at concentrations equal to or higher than the MIC was spread onto MacConkey agar plates and incubated at 37°C for 24 h. The MBC was defined as the lowest BP concentration that killed more than 99.9% of the initially inoculated bacteria.

### RT-qPCR analysis

RT-qPCR was performed as previously described (Chi et al., 2024). Briefly, total RNA was extracted from chicken liver tissues or cell samples using TRNzol reagent (TIANGEN, Beijing, China), following the manufacturer's protocols. Reverse transcription was performed with the HiScript III RT SuperMix (Vazyme Biotech, Nanjing, China). Quantitative PCR (qPCR) was subsequently carried out using 2 × SupRealQ Ultra Hunter SYBR qPCR Master Mix (U+) (Vazyme Biotech, Nanjing, China) on a LightCycler system (Roche, Switzerland). The qPCR program was performed as follows: initial denaturation at 95°C for 30 s, followed by 40 cycles of 95°C for 10 s and 60°C for 30 s. The relative expression levels of target genes were calculated using the 2^-ΔΔCT^ method, with β-actin serving as the internal control. All primer sequences are listed in Table 1.

**Table 1:** The sequence of primers used in this study.

Gene	Primer sequences (5′−3′)		Product size
IL-1β	F: TTCATTACCGTCCCGTTG	R: GCTTTTATTTCTCCAGTCACA	121 bp
IL-6	F: AAATCCCTCCTCGCCAATCT	R: CCCTCACGGTCTTCTCCATAAA	106 bp
IL-10	F: AGATGCTGCGCTTCTACACA	R: CCCATGCTCTGCTGATGACT	76 bp
IL-17A	F: GATGCTGGATGCCTAACCCA	R: TGTGGTCCTCATCGATCCTGT	154 bp
IL-18	F: AGAGATCGCTGTGTGTGCAG	R: CATCGCATTCCAGCTCATCA	77 bp
IL8L1	F: ATGGCTGGAGCAAAAGGTATGG	R: CAGACACACTTCTCTGCCATCT	186 bp
IL8L2	F: TGCTCTGTCGCAAGGTAGGA	R: GGTCCAAGCACACCTCTCTT	188 bp
CCL4	F: AAAGCCTGCCATCATCTTCATCA	R: TTGACGCTCTGCAGGTATCTC	93 bp
CXCL13	F: GAGGAAAGAGATCATCCTCACCC	R: AAGGGACACAGGTCCTGCTA	192 bp
CXCL14	F: CTTAGCCAGTGCAGAAGGAGT	R: CACTGTGTTCTGGCGTTTGG	193 bp
CCL20	F: CAGTACCTCCCAGGCACAAA	R: AATGACCTTCCGAGGCAGAC	76 bp
CX3CL1	F: CTATGGCTGGAGGGCAACC	R: CAGTCTCGCGGTAGCTCTTT	105 bp
iNOS	F: CCTGGAGGTCCTGGAAGAGT	R: CCTGGGTTTCAGAAGTGGC	82 bp
PKM2	F: AAGCAGCAGCAGGAGACAC	R: GTCTGGATGAAAGCGGTCCC	75 bp
HIF-1α	F: CGTCACCGACAAGAAGAGGATT	R: CTTGTCAAGGTGGGCACTCA	151 bp
PTGES	F: CACAGCTCCAAGGAAGAGGA	R: AGGCTCAGGAAGAAGGCATT	153 bp
COX-2	F: TGTCCTTTCACTGCTTTCCAT	R: TTCCATTGCTGTGTTTGAGGT	84 bp
β-actin	F: GAGAAATTGTGCGTGACATCA	R: CCTGAACCTCTCATTGCCA	152 bp

### Western blotting

Tissue and cell samples were lysed in RIPA buffer with protease inhibitors, and subjected to Western blot analysis as previously described (Zhou et al., 2026). Briefly, protein samples were separated by 10% SDS-PAGE, and transferred onto nitrocellulose membranes. The membranes were blocked with 5% non-fat milk for 1 h at room temperature, then incubated with primary antibodies overnight at 4°C. After washing with TBST, membranes were incubated with HRP-conjugated secondary antibodies for 1 h at room temperature. Protein bands were visualized using enhanced chemiluminescence (ECL) reagent and detected with an Odyssey XF Imaging System (LI-COR Bioscience, USA).

### Cell viability assay

Cell viability was assessed using CCK-8 assay as previously described (Ma et al., 2022). Briefly, HD11 cells were seeded into 96-well plates at a density of 2 × 10^4^ cells per well and incubated overnight. Following treatment with BP, 10 μL of CCK-8 solution (Beyotime) was added to each well, and the plates were incubated at 37°C for an additional 2 h. Absorbance was measured at 450 nm using a microplate reader (Tecan Infinite M200 Pro, Switzerland), and cell viability was calculated relative to control groups.

### Determination of ROS levels

Intracellular ROS levels were measured using the ROS Assay Kit (Beyotime) according to the manufacturer's instructions. Briefly, cells cultured in 6-well plates were incubated with 10 μM DCFH-DA (diluted in serum-free medium) at 37°C for 20 min in the dark. After incubation, cells were washed three times with PBS to remove excess probe. Fluorescence was then visualized using a fluorescence microscope (Nikon Eclipse Ti-E, Japan).

### Statistical analysis

Multiple comparison analysis of the data was performed using GraphPad Prism version 9.5 via one-way analysis of variance (ANOVA) followed by post-hoc Tukey’s test. Data are represented as mean ± SD (standard deviation). Differences were considered statistically significant with *p* < 0.05.

## Results

### In vitro antibacterial activity of BP

To evaluate the antibacterial activity of BP, the MIC and MBC of BP against *E. coli, Salmonella*, and *Staphylococcus aureus* were determined using the two-fold dilution method. The results showed that the MIC values of BP against *E. coli* O78, *E. coli* O1, *Salmonella Enteritidis*, and *Salmonella Typhimurium* ranged from 1.9531 µg/mL to 3.9062 µg/mL, whereas the MIC against *Staphylococcus aureus* ranged from 0.9765 µg/mL to 1.9531 µg/mL. The MBC of BP ranged from 7.8125 to 15.625 µg/mL for *E. coli* O78 and *E. coli* O1, from 3.9062 to 7.8125 µg/mL for *Salmonella Enteritidis* and *Salmonella Typhimurium*, and from 1.9531 to 3.9062 µg/mL for *Staphylococcus aureus* (Table 2).

**Table 2:** MIC and MBC of BP against different bacterial strains.

Strain	MIC	MBC
*E.coli* O78	1.9531 µg/mL - 3.9062 µg/mL	7.8125 µg/mL - 15.625 µg/mL
*E.coli* O1	1.9531 µg/mL - 3.9062 µg/mL	7.8125 µg/mL - 15.625 µg/mL
*Salmonella Enteritidis*	1.9531 µg/mL - 3.9062 µg/mL	3.9062 µg/mL - 7.8125 µg/mL
*Salmonella Typhimurium*	1.9531 µg/mL - 3.9062 µg/mL	3.9062 µg/mL - 7.8125 µg/mL
*Staphylococcus aureus*	0.9765 µg/mL - 1.9531 µg/mL	1.9531 µg/mL - 3.9062 µg/mL

**Fig. 1:**
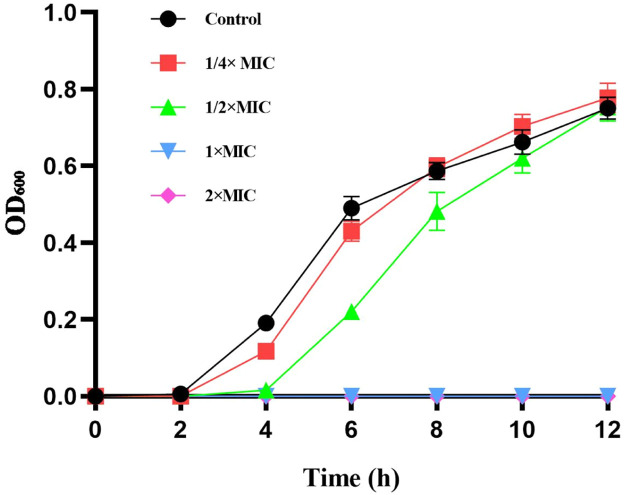
**Growth curves of *E. coli* O78 treated with different concentrations of BP.***E. coli* O78 suspension at 1.0 × 10^5^ CFU/mL was added to LB broth containing various concentrations of BP, and the OD value at 600 nm was measured every 2 h.

To further investigate the inhibitory effect, the impact of BP at sub-inhibitory and inhibitory concentrations (1/4 ×, 1/2 ×, 1 ×, and 2 × MIC) on the growth kinetics of *E. coli* O78 was assessed. The results demonstrated a dose-dependent inhibitory effect on bacterial proliferation. At concentrations of 1 × MIC and above, bacterial growth was completely suppressed throughout the 12-hour testing period. In contrast, cultures treated with sub-inhibitory concentrations (1/4 × and 1/2 × MIC) exhibited a delayed growth phase; although initial growth was suppressed, the optical density reached levels comparable to the untreated control by 12 hours (Fig. 1). These findings confirm that BP effectively inhibits the growth of *E. coli* O78 in a concentration-dependent manner.

### BP significantly reduces E. coli load and prevents hepatic inflammation in infected broilers

**Fig. 2:**
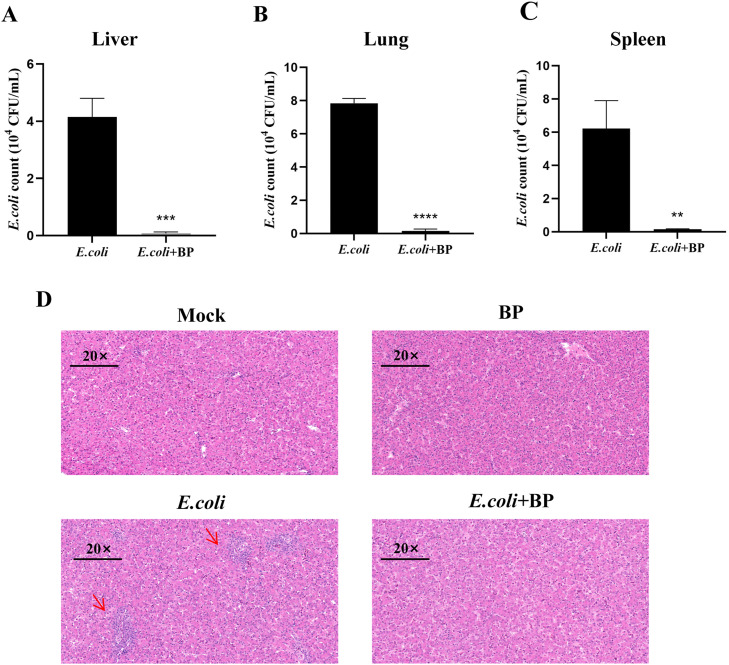
**BP reduces *E. coli* load and prevents hepatic inflammation in infected broilers.** (A-C) Livers, lungs, and spleens were collected from broilers in the *E. coli* and *E. coli* + BP groups, and bacterial load was determined by colony counting. (D) Livers were collected from broilers in the Mock, BP, *E. coli* and *E. coli* + BP groups, and histological lesions were examined microscopically after H&E staining. Data are represented as mean ± SD; Shown are representative data from three biologically independent experiments; **P < 0.01, ***P < 0.001, ****P < 0.0001.

To evaluate the effect of BP on *E. coli* colonization in visceral tissues of white-feathered broilers, liver, lung, and spleen samples were collected from birds in the *E. coli* group and the *E. coli* + BP group at 72 hours post-infection, and bacterial loads were subsequently quantified. Compared with the *E. coli* group, the *E. coli* + BP group exhibited significantly reduced bacterial loads in the liver, lung, and spleen tissues (Fig. 2A-C).

To assess the protective effect of BP against *E. coli*-induced liver injury, histopathological examination was performed on liver tissues from infected birds. Extensive inflammatory cell infiltration was observed in the portal area of the liver in the *E. coli* group (indicated by red arrows). In contrast, the *E. coli* + BP group displayed intact cellular architecture with no evidence of inflammatory cell infiltration (Fig. 2D). Together, these findings demonstrate that BP confers significant protective effects against *E. coli* infection in broilers by reducing bacterial colonization and alleviating tissue inflammation.

### BP attenuates hepatic expression of inflammatory cytokine and chemokine mRNAs

**Fig. 3:**
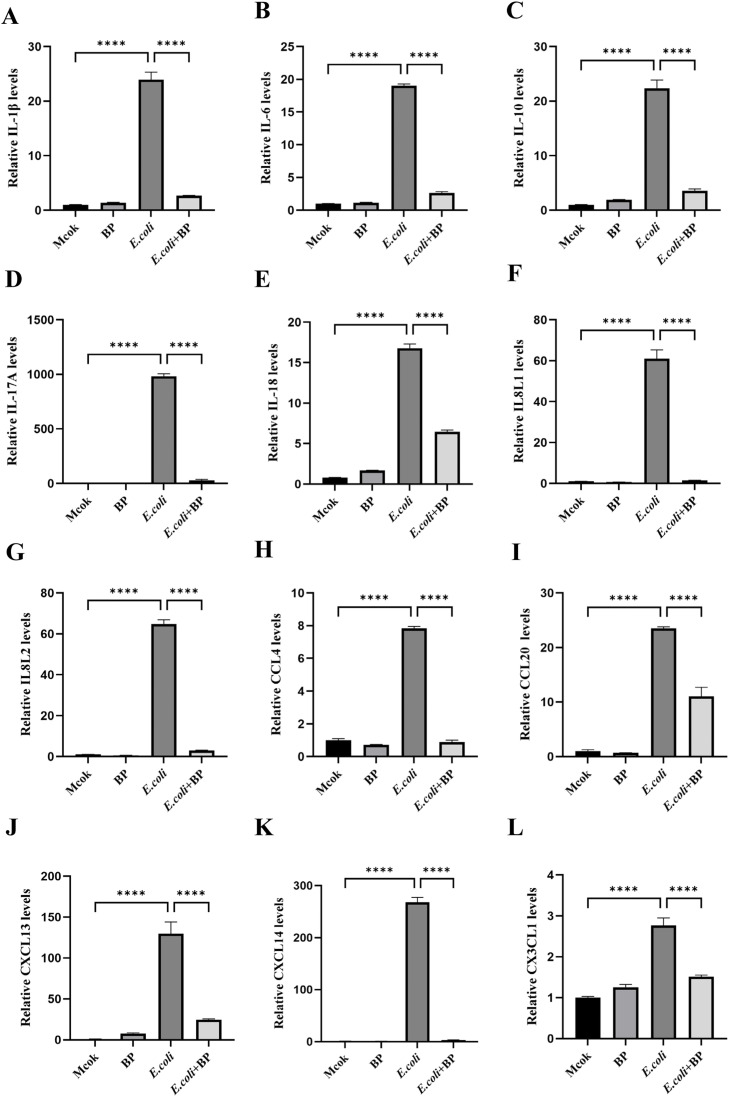
**BP attenuates expression of inflammatory cytokines and chemokines in the liver of *E. coli*-infected broilers.** (A-E) Hepatic tissues were collected from broilers in the Mock, BP, *E. coli* and *E. coli* + BP groups, and the mRNA expression levels of IL-1β (A), IL-6 (B), IL-10 (C), IL-17A (D), and IL-18 (E) were determined by RT-qPCR. (F-L) Hepatic tissues were collected from broilers in the Mock, BP, *E. coli* and *E. coli* + BP groups, and the mRNA expression levels of IL8L1 (F), IL8L2 (G), CCL4 (H), CCL20 (I), CXCL13 (J), CXCL14 (K), and CX3CL1 (L) were determined by RT-qPCR. Data are represented as mean ± SD; Shown are representative data from three biologically independent experiments; ****P < 0.0001.

The expression levels of inflammatory cytokines can directly reflect the inflammatory status in organisms. To evaluate the effect of BP on hepatic inflammation, the mRNA expression levels of inflammatory cytokines and chemokines were examined in each experimental group. Compared with the Mock group, the *E. coli* group exhibited extremely significant upregulation of IL-1β, IL-6, IL-10, IL-17A, and IL-18 mRNA expression levels (Fig. 3A-E). In contrast, treatment with BP resulted in extremely significant downregulation of these cytokine transcripts relative to the *E. coli* group (Fig. 3A-E).

Similarly, the expression levels of chemokines were assessed. In the *E. coli* group, the mRNA expression levels of IL8L1, IL8L2, CCL4, CCL20, CXCL13, CXCL14, and CX3CL1 were extremely significantly increased compared with the Mock group (Fig. 3F-L). In contrast, treatment with BP significantly suppressed the expression of all tested chemokine transcripts compared with the *E. coli* group (Fig. 3F-L). Collectively, these results demonstrate that BP treatment effectively attenuates the *E. coli*-induced hepatic inflammatory response by suppressing the expression of pro-inflammatory cytokines and chemokines at the transcriptional level.

### BP suppresses in vitro expression of inflammatory cytokine and chemokine mRNAs

**Fig. 4:**
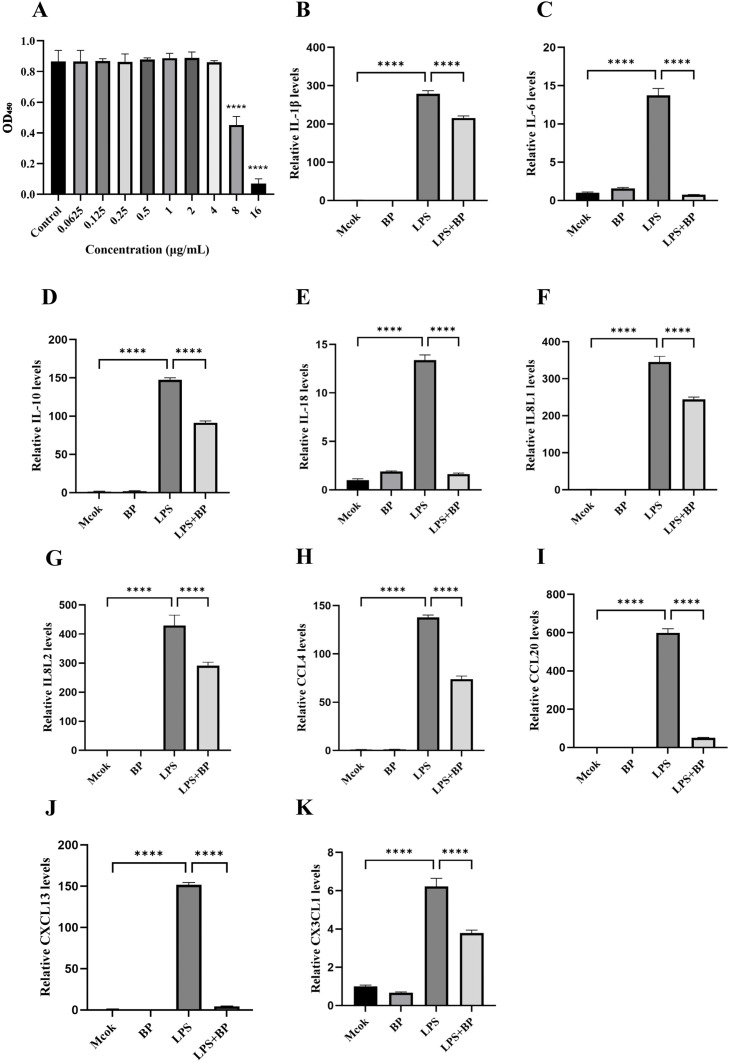
**BP suppresses expression of inflammatory cytokines and chemokines in LPS-stimulated HD11 cells.** (A) Cell viability was measured after HD11 cells were treated with various concentrations of BP for 24 h. (B-E) After pretreatment with BP for 2 h, HD11 cells were stimulated with LPS (1 μg/mL) for 4 h. The mRNA expression levels of IL-1β (B), IL-6 (C), IL-10 (D), and IL-18 (E) were then determined by RT-qPCR. (F-K) HD11 cells were treated as described in (B-E), and the mRNA expression levels of IL8L1 (F), IL8L2 (G), CCL4 (H), CCL20 (I), CXCL13 (J), and CX3CL1 (K) were determined by RT-qPCR. Data are represented as mean ± SD; Shown are representative data from three biologically independent experiments; ****P < 0.0001.

To further investigate the direct anti-inflammatory effect of BP at the cellular level, we evaluated its impact on inflammatory cytokine and chemokine expression in HD11 cells. Prior to these experiments, the cytotoxicity of BP was assessed using the CCK-8 assay. The results showed that BP had no effect on cell viability at concentrations of 4 μg/mL or lower, whereas cell viability was reduced at concentrations above 4 μg/mL (Fig. 4A). Based on this finding, a concentration of 4 μg/mL was selected for subsequent experiments.

As shown in Fig. 4B-E, BP alone did not significantly alter the expression of pro-inflammatory cytokines in the absence of LPS stimulation compared with the untreated control. However, treatment with BP significantly suppressed the expression of these cytokines induced by LPS stimulation in HD11 cells. Specifically, the mRNA expression levels of IL-1β, IL-6, IL-10, and IL-18 were markedly elevated in the LPS group, whereas BP treatment resulted in significant downregulation of these cytokine transcripts (Fig. 4B-E). Similarly, the expression of chemokines was assessed. Compared with the LPS group, the mRNA expression levels of IL8L1, IL8L2, CCL4, CCL20, CXCL13, and CX3CL1 were extremely significantly downregulated in the BP-treated group (Fig. 4F-K). Together, these findings demonstrate that BP exerts direct anti-inflammatory effects on HD11 cells by attenuating the expression of key inflammatory cytokines and chemokines at the transcriptional level.

### BP attenuates hepatic and cellular expression of pro-inflammatory mediator mRNAs in vivo and in vitro

**Fig. 5:**
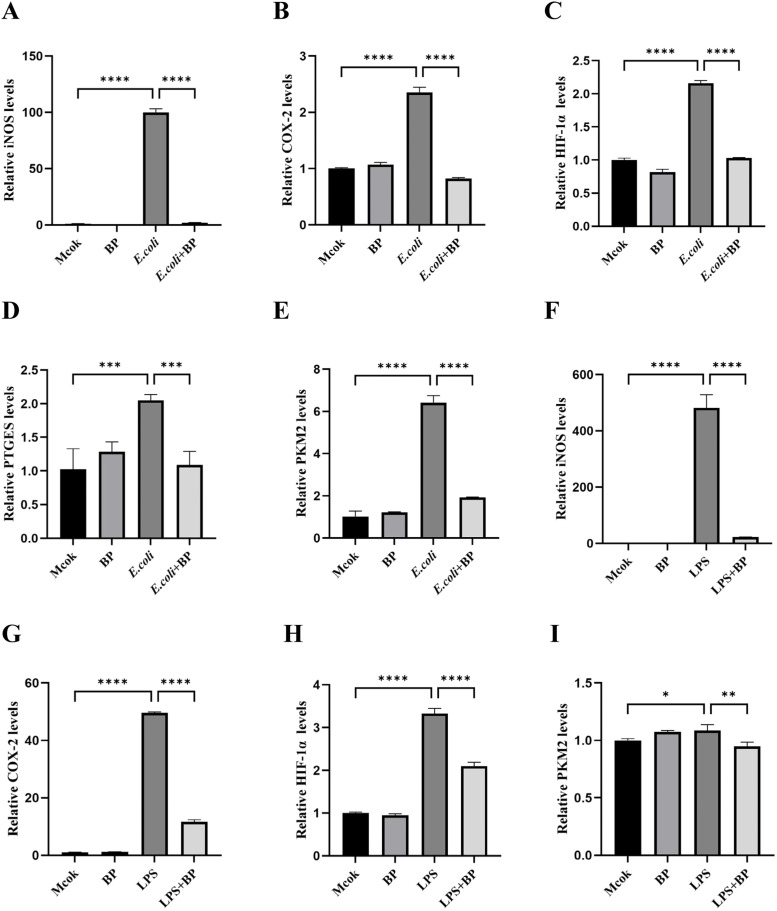
**BP inhibits hepatic and cellular expression of pro-inflammatory mediators both *in vivo* and *in vitro*.** (A-E) Hepatic tissues were collected from broilers in the Mock, BP, *E. coli* and *E. coli* + BP groups, and the mRNA expression levels of iNOS (A), COX-2 (B), HIF-1α (C), PTGES (D), and PKM2 (E) were determined by RT-qPCR. (F-I) After pretreatment with BP for 2 h, HD11 cells were stimulated with LPS (1 μg/mL) for 4 h. The mRNA expression levels of iNOS (F), COX-2 (G), HIF-1α (H), and PKM2 (I) were then determined by RT-qPCR. Data are represented as mean ± SD; Shown are representative data from three biologically independent experiments; *P < 0.05, **P < 0.01, ***P < 0.001, ****P < 0.0001.

To further evaluate the anti-inflammatory efficacy of BP, the mRNA expression levels of key pro-inflammatory mediators were examined both *in vivo* and *in vitro*. In the *in vivo* experiment, the mRNA expression levels of iNOS, COX-2, HIF-1α, PTGES, and PKM2 were significantly elevated in the *E. coli* group compared with the Mock group (Fig. 5A-E). In contrast, BP treatment resulted in significant downregulation of these transcripts relative to the *E. coli* group (Fig. 5A-E).

Consistent with these findings, the *in vitro* experiment revealed similar results. In HD11 cells, LPS stimulation led to significant upregulation of iNOS, COX-2, HIF-1α and PKM2 mRNA expression compared with the Mock group (Fig. 5F-I). Conversely, treatment with BP significantly suppressed the expression of all tested pro-inflammatory mediators in LPS-stimulated cells (Fig. 5F-I). Together, these findings suggest that BP exerts its anti-inflammatory effects by suppressing the transcriptional upregulation of key pro-inflammatory mediators in response to bacterial challenge.

### BP inhibits activation of the NF-κB and MAPK signaling pathways in vivo and in vitro

**Fig. 6:**
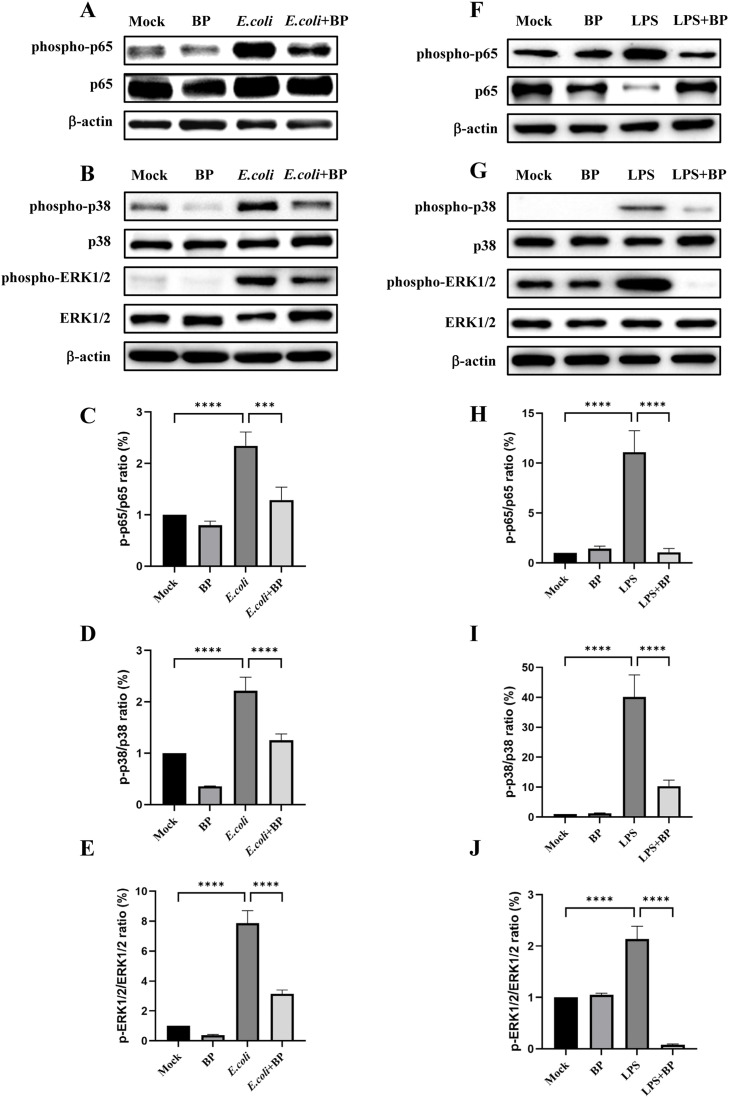
**BP inhibits activation of the NF-κB and MAPK signaling pathways both *in vivo* and *in vitro*.** (A, B) Hepatic tissues were collected from broilers in the Mock, BP, *E. coli* and *E. coli* + BP groups, and the phosphorylation levels of NF-κB p65 (A), p38 and ERK1/2 (B) were examined by Western blotting. (C-E) The phosphorylation levels of p65, p38, and ERK1/2 in (A, B) were quantified by densitometry and normalized to their respective total protein levels. (F, G) After pretreatment with BP for 2 h, HD11 cells were stimulated with LPS (1 μg/mL) for 4 h. The phosphorylation levels of NF-κB p65 (F), p38 and ERK1/2 (G) were then examined by Western blotting. (H-J) The phosphorylation levels of p65, p38, and ERK1/2 in (F, G) were quantified by densitometry and normalized to their respective total protein levels. Data represent mean ± SD from three independent experiments; ***P < 0.001, ****P < 0.0001.

To elucidate the mechanisms underlying the anti-inflammatory effects of BP, the activation status of the NF-κB and mitogen-activated protein kinase (MAPK) signaling pathways was assessed in both hepatic tissues and HD11 cells. As shown in Fig. 6A-E, infection with *E. coli* led to robust increases in the phosphorylation levels of p65, p38, and ERK1/2 in the liver compared with Mock controls. Administration of BP effectively reversed this effect, with markedly diminished phosphorylation of these signaling molecules observed in the *E. coli* + BP group.

Similar results were obtained *in vitro*. Upon LPS stimulation, HD11 cells displayed pronounced phosphorylation of p65, p38, and ERK1/2 relative to untreated controls. However, co-treatment with BP markedly attenuated LPS-induced phosphorylation of these signaling mediators (Fig. 6F-J). Together, these results suggest that BP exerts anti-inflammatory activity by inhibiting the NF-κB and MAPK signaling pathways in response to bacterial infection or LPS stimulation.

### BP reduces intracellular ROS levels in LPS-stimulated cells

**Fig. 7:**
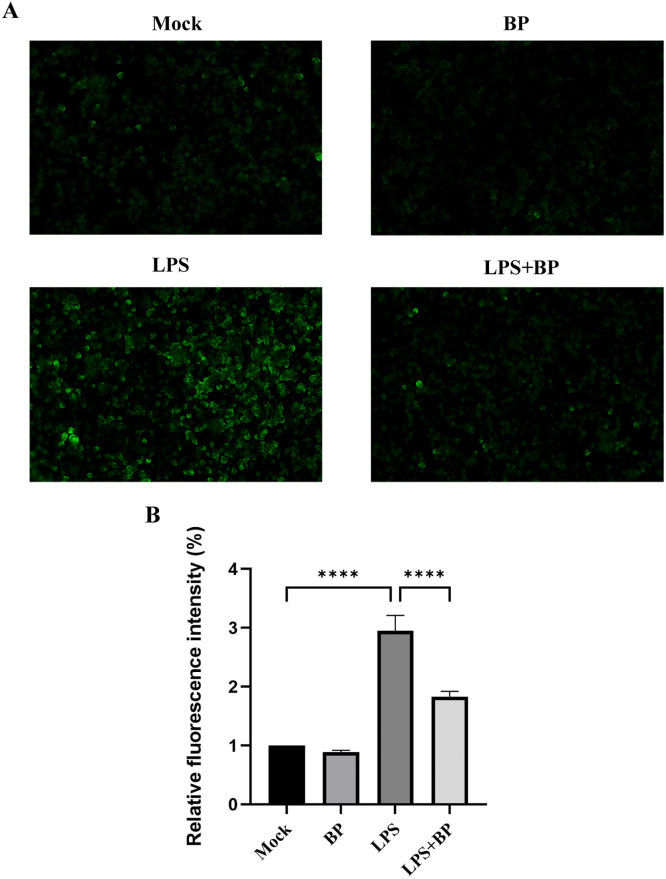
**BP reduces ROS levels in LPS-stimulated HD11 cells.** (A) HD11 cells were pretreated with BP for 2 h and then stimulated with LPS (1 μg/mL) for 4 h. Subsequently, the cells were loaded with the DCFH-DA fluorescent probe, and fluorescence intensity was visualized under an inverted fluorescence microscope. (B) Fluorescence intensity in (A) was quantified using ImageJ software. Data represent mean ± SD from three independent experiments; ****P < 0.0001.

To further investigate the potential mechanism underlying the anti-inflammatory effects of BP, the production of ROS was assessed in HD11 cells using fluorescence-based detection. Under control conditions, HD11 cells exhibited weak fluorescent signal. Upon stimulation with LPS, a pronounced increase in fluorescence intensity was observed (Fig. 7A and B), indicating elevated ROS production. In contrast, treatment with BP markedly diminished LPS-induced fluorescence (Fig. 7A and B), suggesting that BP effectively suppresses ROS generation in activated HD11 cells. These results indicate that BP exerts antioxidant activity by reducing LPS-induced ROS production, which may contribute to its anti-inflammatory effects.

## Discussion

*E. coli* is ubiquitously distributed in the avian intestinal tract, with the majority of strains being non-pathogenic and constituting an integral component of the intestinal microbiota. However, certain pathogenic strains can cause extraintestinal systemic infections in birds, a clinical condition defined as colibacillosis (Joseph et al., 2023). In response to the implementation of policies restricting the use of antibiotics in animal feed, polyphenols, as plant secondary metabolites with antioxidant, antibacterial and anti-inflammatory properties, have received extensive attention in the poultry industry. China possesses abundant bamboo resources, accounting for approximately one-quarter of the world's total bamboo forest area. As a renewable and widely available natural resource, bamboo represents a sustainable source of bioactive polyphenols, making bamboo-derived polyphenols particularly attractive for large-scale application in the poultry industry.

In the present study, *in vitro* MIC and MBC assays were performed to evaluate the bacteriostatic and bactericidal activities of BP. The results demonstrated that BP exhibited potent inhibitory and bactericidal effects against both Gram-positive and Gram-negative bacteria. These findings are consistent with previous reports showing that polyphenol-rich red wine extracts possess strong bactericidal activity against *E. coli* and *Staphylococcus aureus* (Chala et al., 2024). Furthermore, analysis of the effects of BP on the growth kinetics of *E. coli* O78 revealed a concentration-dependent bacteriostatic effect. Accumulating evidence suggests that polyphenolic compounds exert antimicrobial activity by binding to bacterial proteins, including adhesins, enzymes, and membrane transporters, thereby inactivating these target proteins (Makarewicz et al., 2021).

Following infection with APEC, the pathogen colonizes multiple vital tissues and organs, where it undergoes extensive proliferation and induces severe pathological lesions. Previous studies have demonstrated that APEC infection causes pronounced perihepatitis and pericarditis in broilers (Zhang et al., 2025). In the present study, we observed that the *E. coli* burden in the liver, spleen, and lung was significantly reduced in the *E. coli* + BP group compared with the *E. coli* group. These findings are consistent with earlier reports showing that curcumin, a polyphenolic compound, inhibits the growth of methicillin-resistant *Staphylococcus aureus* (MRSA) in the liver and reduces bacterial load (Witika et al., 2021).

Colonization of host tissues by APEC inevitably triggers extensive inflammatory cell infiltration, a finding corroborated by our histopathological examination. Marked inflammatory cell infiltration was observed in the portal areas of liver sections from the *E. coli* group. In contrast, no such extensive infiltration was detected in the *E. coli* + BP group. This observation suggests that BP may mitigate inflammatory cell accumulation, thereby alleviating histopathological damage in infected tissues.

Previous studies have established that infection with APEC triggers extensive immune cell infiltration in host tissues, a phenomenon attributed to the release of large quantities of endotoxins, such as LPS, during bacterial proliferation and colonization (Abdelhamid et al., 2024). Upon binding to Toll-like receptor 4 (TLR4) on the cell surface, LPS activates TLR4, which subsequently transduces intracellular signals to recruit MyD88 and activate the NF-κB and MAPK signaling pathways. This cascade ultimately induces the robust secretion of inflammatory cytokines, chemokines, and pro-inflammatory mediators. When produced in moderate amounts, these cytokines play a pivotal role in innate immunity. However, excessive immune cell accumulation in host tissues is typically driven by the overexpression of these mediators, which can lead to severe tissue damage and high mortality rates in affected poultry.

Resveratrol, a well-characterized non-flavonoid polyphenolic compound, has been shown to suppress LPS-induced inflammation in RAW264.7 cells by downregulating the expression of pro-inflammatory cytokines, including IL-1 and IL-6 (Monmai et al., 2023). Similarly, a study investigating the effects of resveratrol on LPS-induced apoptosis and inflammatory responses in HT-29 cells confirmed that resveratrol exerts anti-inflammatory effects by reducing the expression of pro-inflammatory cytokines such as IL-6 and IL-18 (Zhao et al., 2024). In the present study, both the *E. coli* group and the LPS group exhibited excessive mRNA expression of inflammatory cytokines, chemokines, and pro-inflammatory mediators, whereas treatment with BP led to extremely significant reductions in the expression levels of these factors. Our findings further support the notion that polyphenolic compounds exert anti-inflammatory effects through the downregulation of pro-inflammatory cytokine expression at the transcriptional level.

In response to inflammatory stimuli such as LPS, the binding of LPS to TLR4 triggers the activation of multiple downstream signaling cascades, including the NF-κB and MAPK pathways. The transcription factor NF-κB plays a central role in regulating gene expression during immune and inflammatory responses (Hayden et al., 2008). In unstimulated cells, NF-κB resides in the cytoplasm in an inactive form, bound to its inhibitory protein IκB. Upon exposure to inflammatory stimuli such as LPS, IκB kinase (IKK) phosphorylates IκB, leading to its ubiquitination and subsequent proteasomal degradation (Xue et al., 2008). This process results in the phosphorylation and release of NF-κB. Subsequently, NF-κB translocates to the nucleus to initiate the transcription of inflammatory mediators, including iNOS and COX-2, thereby amplifying the inflammatory response (Liu et al., 2023). The MAPK family comprises three major subfamilies: extracellular signal-regulated kinase (ERK), p38, and c-Jun N-terminal kinase (JNK), all of which play critical roles in regulating inflammatory gene expression. While NF-κB serves as a central transcription factor mediating the expression of pro-inflammatory cytokines and enzymes, MAPK pathways can also promote inflammation through alternative transcription factors, most notably activator protein-1 (AP-1). Importantly, AP-1 activation can occur independently of NF-κB, representing a parallel pathway through which MAPK signaling contributes to inflammation. Indeed, studies have demonstrated that inhibition of MAPK suppresses AP-1 activity and downstream inflammatory gene expression without necessarily affecting NF-κB activation (Niu et al., 2025).

In the present study, we observed that *E. coli* infection *in vivo* and LPS stimulation *in vitro* both led to significantly increased phosphorylation of p65 (NF-κB subunit), p38, and ERK1/2, indicating concurrent activation of both NF-κB and MAPK signaling pathways. Notably, treatment with BP markedly attenuated the phosphorylation of these key signaling molecules. Given that p38 and ERK are upstream activators of AP-1, we speculate that BP not only inhibits the canonical NF-κB pathway but also suppresses MAPK-dependent AP-1 activation, thereby reducing the transcription of inflammatory cytokines, chemokines, and pro-inflammatory enzymes. This dual inhibition may contribute to the robust anti-inflammatory effects observed both *in vivo* and *in vitro*.

In addition to the activation of NF-κB and MAPK, ROS have been recognized as critical upstream signaling molecules that modulate inflammatory responses (Kim et al., 2007). Under physiological conditions, intracellular ROS levels are maintained at low concentrations by endogenous antioxidant systems. However, upon stimulation with LPS or bacterial infection, NADPH oxidases and mitochondrial electron transport chains become activated, leading to a burst of ROS production. Elevated ROS levels can promote the activation of both NF-κB and MAPK signaling pathways through redox-sensitive mechanisms (Bellanti et al., 2025). In the present study, LPS stimulation of HD11 cells resulted in a pronounced increase in ROS production, whereas treatment with BP significantly attenuated this increase. Given the established role of ROS in promoting NF-κB and MAPK activation, we speculate that BP may exert its anti-inflammatory effects, at least in part, by reducing ROS generation, thereby suppressing the downstream activation of these critical signaling pathways and the subsequent expression of pro-inflammatory mediators. This speculation is supported by a recent study showing that SIRT3-mediated mitochondrial fatty acid oxidation protects against hepatic oxidative damage in laying hens (Cao et al., 2026), highlighting the potential link between ROS regulation, mitochondrial function, and inflammatory injury.

In addition to the NF-κB/MAPK pathways and ROS, the autophagy-lysosome system has emerged as a critical player in pathogen-induced tissue injury. A recent study by Huang et al. demonstrated that avian pathogen infection blocks autophagic flux and impairs lysosomal function via inhibition of the AMPK-TFEB signaling pathway, thereby exacerbating kidney injury in chicks (Huang et al., 2026). Given the established crosstalk between autophagy and inflammation, we speculate that BP may exert its protective effects not only through inhibition of NF-κB/MAPK signaling and ROS reduction, but also potentially through modulation of the AMPK-TFEB-autophagy axis. This hypothesis needs further investigation in future studies.

Several limitations of this study should be acknowledged. First, the APEC infection model was established via intraperitoneal injection, which bypasses the natural respiratory route. Thus, our findings primarily reflect the protective effect of BP against systemic bacterial dissemination and hepatic inflammation, rather than its efficacy against respiratory mucosal infection. Second, a positive control group was not included, precluding direct comparison of BP's relative efficacy with standard anti-inflammatory agents. Nonetheless, the consistent results across *in vivo* and *in vitro* experiments, including suppression of inflammatory cytokines, inhibition of NF-κB/MAPK phosphorylation, and attenuation of histopathological damage and ROS production, provide robust evidence for the anti-inflammatory activity of BP. Future studies employing intratracheal infection models and incorporating positive controls are necessary to further validate and evaluate the therapeutic potential of BP.

## Conclusion

In summary, BP exhibits potent antibacterial and anti-inflammatory activities against APEC infection both *in vivo* and *in vitro*. It inhibits bacterial proliferation, reduces organ colonization, and alleviates tissue inflammation by suppressing NF-κB and MAPK signaling pathways, downregulating pro-inflammatory mediators, and attenuating ROS production. These findings support BP as a promising alternative to antibiotics for the management of colibacillosis in poultry.

## Ethics statement

The animal protocol used in this study was approved by the Research Ethics Committee of Fujian Agriculture and Forestry University (Permit Number PZCASFAFU25143). All animal experiments were performed in accordance with the Regulations for the Administration of Affairs Concerning Experimental Animals of the Ministry of Science and Technology of the People’s Republic of China.

## Funding

This work was supported by Major Science and Technology Project of Fujian Province of China (2024NZ029018), 10.13039/501100001809National Natural Science Foundation of China (32573353), and Science and Technology Innovation Project of Fujian Agriculture and Forestry University (KFB23094).

## Authorship contribution

Song Wang and Xiaojuan Chi conceived the project and supervised the overall study. Yuzhang Chen and Song Wang designed the study. Yuzhang Chen, Jie Zeng, Lu Liu, Ning Li, Mengyao Liu, Kaicheng Zhang, Zexi Zhang, and Xiaojuan Chi performed the experiments and data analysis. Bingjie Zou performed sample collection and data analysis. Yuzhang Chen drafted the original manuscript, and Song Wang critically revised and edited the manuscript. All authors read and approved the final manuscript.

## Disclosures

The authors declare that they have no known competing financial interests or personal relationships that could have appeared to influence the work reported in this paper.

## Data Availability

All data generated or analyzed during this study are included in this published article.
